# When technology meets judgment: outcome of football referees’ disciplinary decision-making after the implementation of VAR in the English Premier League

**DOI:** 10.3389/fpsyg.2026.1769008

**Published:** 2026-03-19

**Authors:** Bjørn Tore Johansen, Per Thomas Byrkjedal, Are Walman Johnsen, Adrian Galdal Madsen, Erik Kaarstein

**Affiliations:** 1 Department of Sport Science and Physical Education, University of Agder, Kristiansand, Norway; 2 School of Sport Sciences, The Artic University of Norway, Tromsø, Norway

**Keywords:** difference-in-differences (DiD), English Premier League, referee decision-making, technology, Video Assistant Referee (VAR)

## Abstract

**Introduction:**

Video Assistant Referee (VAR) is a decision-support system that helps referees make more accurate decisions and eliminate clear and obvious errors. However, the implementation of VAR in world football has been one of the most significant and controversial technological procedures used.

**Methods:**

This study examines how the introduction of VAR has affected refereeing decisions in the English Premier League (EPL) by examining the number of red cards and penalties per match. Yellow cards were also implemented in the analyses as a possible hypothesis-generating cause. Using match-level data from the 2018/19 season (without-VAR) and the 2023/24 season (with-VAR), and using the English Championship as a control group, we apply a difference-in-differences (DiD) analysis to estimate the contributive effect of VAR’s impact on three key outcomes: red cards, penalties and yellow cards awarded per match.

**Results:**

The results suggest a statistically significant increase in all three outcomes in the Premier League following the introduction of VAR, compared to the Championship where VAR was not implemented. The increase in red cards and penalties, both explicitly governed by the VAR protocol, likely reflects improved identification of clear and obvious errors. The observed rise in yellow cards suggests that VAR may exert an indirect influence on referee’s behaviour, although this finding needs further investigation to be verified.

**Discussion:**

Notably, the estimates found in current study reflect only differences between the Premier League and the Championship and no further definitive causal effects should be drawn. This study contributes to the ongoing debate about VAR’s role and potential impact on football referees’ disciplinary decision-making behaviour and provides a foundation for further empirical research on the broader implications of VAR in football.

## Introduction

The Video Assistant Referee (VAR) system represents a major institutional and regulatory change in elite football officiating, intended to support referees in correcting “clear and obvious errors” and thereby improve decision accuracy in match-decisive situations (FIFA, 2016; Premier League, 2025; Zglinski, 2020). Refereeing in professional football is performed under high perceptual, temporal, and social constraints, where decisions must often be made within seconds and under intense scrutiny from players, coaches, and spectators (Mascarenhas et al., 2008; Lex et al., 2015). In this context, decision-aid technologies have increasingly been adopted across sports to reduce error and enhance legitimacy, yet their introduction may also reshape behavioural norms, authority relations, and the pace and experience of competition (Nagle et al., 2024; Scanlon et al., 2022). Still, in football, and in different game sports, use of technological officiating aids to support the referees (Kolbinger and Lames, 2017), and the VAR system is one example of how technology is used to assist referees’ decision-making, although its impact on disciplinary actions remains unknown (Spitz et al., 2021) and needs to be further examined.

### VAR protocol and mechanisms of influence

Following IFAB-led trials initiated in 2016, VAR was progressively implemented across top European competitions and was formally approved for broader use in 2018 (FIFA, 2016, 2018). Under the VAR protocol, intervention is restricted to four game-changing scenarios: (i) goals and offences leading to goals, (ii) penalty incidents and offences preceding penalties, (iii) direct red card incidents, and (iv) mistaken identity in disciplinary sanctions (The International Football Association Board (IFAB), 2024). Reviews may occur as a “VAR-only” intervention for factual decisions, or through an on-field review for subjective judgements, with the on-field referee retaining final authority (Spitz et al., 2021). Importantly, several routine decisions, including yellow cards, remain outside the formal remit of VAR (The International Football Association Board (IFAB), 2024; Premier League, 2025). Nevertheless, yellow cards are a salient indicator of referees’ day-to-day threshold setting and match-management, and they may respond indirectly to VAR through behavioural adaptation and institutional recalibration (Nagle et al., 2024; Spitz et al., 2021). For example, heightened scrutiny, perceived accountability, and efforts to maintain control in an environment where “game-changing” incidents are reviewable may alter how borderline conduct is managed even when the specific decision is not itself reviewable (Archibald et al., 2025; Scanlon et al., 2022).

The English Premier League (EPL) is among the most visible professional sport competitions globally, and the league’s prominence implies that officiating changes have considerable sporting and commercial salience (Parnell et al., 2022; Premier League, 2024). The EPL was also the last of the major European leagues to adopt VAR, introducing it in 2019/20 after learning from implementation experiences elsewhere (Bao and Han, 2024; Premier League, 2025). Since adoption, the system has attracted both support and sustained criticism, particularly around perceived inconsistency, disruption of match flow, and the broader legitimacy of officiating outcomes (Bertin et al., 2023; Hamsund and Scelles, 2021; Scanlon et al., 2022). Contemporary accounts further characterize VAR as a socio-technical system, where practical effects emerge through interaction between technology, governance, and on-field decision-making practices (Archibald et al., 2025; Nagle et al., 2024).

### Evidence base and uncertainty on disciplinary outcomes

Empirical research generally indicates that VAR can improve accuracy in reviewable incidents and may reduce certain forms of referee bias, although this may be accompanied by interruptions and altered match experience (Gasparetto and Loktionov, 2023; Scanlon et al., 2022; Spitz et al., 2021). However, the relationship between VAR and decision-making is not uniform across contexts. In “grey-zone” situations—where the Laws of the Game (The International Football Association Board (IFAB), 2024) provide limited objective reference points and interpretation is central—defining a single correct decision is challenging (Spitz et al., 2021). Prominent examples include handball-related incidents, where evolving interpretations and rule amendments have influenced both scrutiny and sanctioning thresholds following the introduction of VAR (Scanlon et al., 2022; Spitz et al., 2021). Such dynamics underscore that VAR may affect not only the correction of clear errors, but also the broader ecology of refereeing—potentially shifting thresholds for sanctioning, player behaviour, and perceptions of fairness.

Previous research (e.g., Han et al., 2020; Bao and Han, 2024; Lago-Peñas et al., 2019, 2021; Spitz et al., 2021) have examined how VAR has influenced the game in a variety of facets. These studies have generated some valuable findings. For instance, the number of offsides, fouls, and yellows cards significantly decreased after the use of VAR in Serie A and Bundesliga; this led to more playing minutes added to the first half and the full game (Lago-Peñas et al., 2019). Spitz et al. (2021) analysed 2,195 soccer matches across 13 countries and a significant increase in decision accuracy was found after VAR intervention. In the Chinese Super League, the number of offsides and fouls also dropped significantly after the implementation of VAR; home team advantage was also inhibited to some extent (Han et al., 2020). Furthermore, a study of the FIFA World Cup revealed that the number of offsides decreased, and the number of penalties increased after the introduction of VAR (Kubayi et al., 2022). Nonetheless, Lago-Peñas et al. (2021) stated that no significant changes were made to the Spanish La Liga match after the introduction of VAR and it is worth mentioning that between-country differences of VAR impact may be attributed to different football cultures, refereeing styles and league styles as Sarmento et al. (2013) found in their comparison study of top leagues in England, Spain, and Italy. In addition, although Tamir and Bar-Eli (2021) pointed out that VAR has been proved to lead to a fairer game Spitz et al. (2021) have indicated that some referees may prefer a harsher punishment watching the video slow motion replay of VAR. One example could be turning a player’s foul that should have resulted in a yellow card into a red card.

Subsequently, evidence on how VAR relates to disciplinary outcomes remains mixed, and cross-league findings do not necessarily generalize to the EPL. Prior work has reported changes in match events following VAR adoption in selected leagues, including reductions in certain match actions such as fouls and yellow cards (Lago-Peñas et al., 2019), while other studies have documented altered technical-tactical patterns and changes in effective playing time associated with VAR interventions (Errekagorri et al., 2020). Indirect links to VAR offenses are highlighted more concretely in studies that refer to yellow cards as a representation at a lower but strategically important sanction level, and are actively used to regulate discipline, intensity, and match flow (Russell et al., 2019). Studies also show that VAR offenses are often followed by increased use of yellow cards, especially in situations related to offenses in and around the penalty area (Samuel et al., 2024).

In the EPL specifically, Bao and Han (2024) did not observe the decline in yellow cards that might be anticipated on the basis of these cross-league reports, raising the possibility that disciplinary dynamics in the EPL differ from those reported elsewhere or that simple before–after comparisons may be confounded by broader temporal changes in playing style, officiating guidelines, and competition context. Therefore, this study investigates whether the implementation of VAR is associated with referees’ decision-making considering disciplinary actions in the EPL. Included in disciplinary actions are yellow cards even if yellow cards are not subject to VAR review (except for mistaken identify). Including yellow cards makes it possible to examine the mechanism behind any changes in red cards and penalty kicks, rather than just observing the disciplinary outcome. Contrasting the study of Bao and Han (2024) we use match-level data from the 2018/19 season (without-VAR) and the 2023/24 season (with-VAR) and using the English Championship as a control group where VAR is not yet implemented.

### Study aim and empirical approach

This study investigates the implementation of VAR in the EPL and how this is associated on match disciplinary outcomes, specifically red cards, penalties, and yellow cards. By including non-reviewable yellow cards, the analysis intends to capture both direct intervention effects and indirect behavioural spillovers.

To strengthen associated inference, we employ a difference-in-differences (DiD) design using the English Championship as a control group. This design isolates VAR-specific effects from general longitudinal trends in English football, contingent upon the satisfaction of the parallel trends assumption.

## Methods

### Research design

The current study examines the impact of the VAR system on referees’ decision-making in the EPL, focusing on changes in the number of red cards, penalties and yellow cards awarded per match. To estimate the potential effect of VAR on disciplinary outcomes in EPL, this study employs a DiD analysis (Caetano and Callaway, 2024; Callaway and Sant’Anna, 2021; Wing et al., 2024) rather than a traditional pre-post analysis. This choice is motivated by the necessity of addressing the fundamental problem of causal analysis: constructing a credible counterfactual scenario to isolate the true effect of the intervention, given the limitations inherent in pre-post comparisons (Angrist and Pischke, 2010). Contrasting previous studies, such as Bao and Han (2024), we utilize match-level data and include the English Championship as a control group where the technology has not yet been implemented. By comparing the change in outcomes in the EPL with the trends observed in the Championship, the DiD design allows us to “differentiate away” time-invariant differences between the leagues and control for common time trends, for example changes in refereeing practices. Consequently, this study approach compares changes in outcomes in the EPL following VAR implementation with corresponding changes in a comparable competition that did not adopt VAR over the same period, thereby isolating VAR-related effects from broader temporal trends affecting English football.

The EPL constitutes the treatment group, with the 2018/19 season representing the final season without VAR and the 2023/24 season representing a period of established VAR use. The English Championship serves as the control group, as VAR was not implemented in this league during either season. Both competitions operate under the same Laws of the Game and within a shared national refereeing structure, supporting their suitability for comparative analysis.

The DiD framework relies on the assumption that, in the absence of VAR, outcomes in the EPL and Championship would have followed similar trajectories over time. To assess the plausibility of this assumption, data from three pre-intervention seasons (2016/17, 2017/18, and 2018/19) are incorporated. These seasons precede VAR adoption in both leagues and provide a basis for evaluating pre-existing trends in referee decision-making.

### Data and variables

Match-level statistics for red cards, penalties and yellow cards were collected from the official EPL website and FotMob (Premier League, 2024; FotMob, 2025). These sources provide consistent and reliable match data for both the EPL and the Championship. The dataset includes all matches from the 2018/19 and 2023/24 seasons for both leagues and additional matches from the 2016/17 to 2018/19 seasons for pre-trend assessment.

Three outcome variables were analyzed: red cards per match, penalties awarded per match, and yellow cards per match. These outcomes differ in their relationship to VAR, as penalties and direct red cards fall within the formal scope of VAR review, whereas yellow cards do not. Inclusion of all three variables allows examination of both direct VAR-related effects and potential indirect changes in referee behaviour.

### Analytical strategy

The analysis proceeds in two stages. First, trends in all outcome variables are examined across the 2016/17–2018/19 seasons to evaluate the plausibility of the parallel trends assumption underlying the DiD design. This assessment is based on examination of pre-intervention outcome trajectories for the EPL and the Championship, using descriptive statistics and graphical inspection to evaluate whether changes evolved similarly prior to VAR implementation.

Second, the main analysis estimates DiD effects by comparing changes in average outcomes between the pre-VAR (2018/19) and post-VAR (2023/24) seasons across the two leagues. The DiD estimator captures differences in outcome changes over time between the treatment and control groups and thus provides an estimate of the association between VAR implementation and referee decision-making outcomes under the maintained identifying assumptions.

Given that the outcomes differ in their relationship to the VAR protocol and because pre-intervention trajectories may vary between leagues, DiD estimates should be interpreted with outcome-specific caution informed by the parallel-trends diagnostics and the formal scope of VAR review.

### Statistical procedures

Data is presented as mean ± SD, unless stated otherwise. Aggregated match-level data were used to compute DiD estimates along with standard error, 95% confidence intervals (CI), *t*-, and *p*-values. These inferential statistics allow for evaluation of the precision and significance of the estimated effects. Descriptive summary statistics for each league and season are provided in Appendix A.

The DiD estimator is calculated using the standard two-group, two-period formulation:


$$\delta_{\mathrm{DD}}=({\overset{-}{Y}}_{B,2}{\overset{-}{Y}}_{B,1})-({\overset{-}{Y}}_{A,2}{\overset{-}{Y}}_{A,1}),$$

where ${\overset{-}{Y}}_{B,2}$ represents the average outcome in the intervention group (EPL) after VAR implementation (2023/24), and ${\overset{-}{Y}}_{B,1}$ represents the average in the same group before VAR (2018/19). Correspondingly, ${\overset{-}{Y}}_{A,2}\;$ and ${\overset{-}{Y}}_{A,1}$ denote the outcome averages in the control group (Championship) for the same two time periods. Table 1 provides a simplified overview of this design structure.

**Table 1:** Simplified representation of the difference-in-difference design.

Unit	Group	Time	Outcome (Y)	Intervention
Premier League 2018/19 (B)	Intervention	Before	Y1	0
Premier League 2023/24 (B)	Intervention	After	Y2	1
Championship 2018/19 (A)	Control	Before	Y1	0
Championship 2023/24 (A)	Control	After	Y2	0

This table provides a simplified overview of the structure underlying the difference-in-difference equation.

## Results

### Parallel trends assessment

To evaluate the plausibility of the parallel trends assumption underpinning the difference-in-differences (DiD) design, trends in red cards, penalties, and yellow cards were examined across the three pre-VAR seasons (2016/17–2018/19) for both the EPL and the Championship. Descriptive values for the pre-intervention period are presented in Table 2. In addition, a graphical illustration of pre-intervention trajectories is provided in the Appendix (see Tables A4, B4, and C4 for yellow cards, red cards, and penalties respectively).

**Table 2:** Average variable per match (2016/17, 2017/18, and 2018/19).

League and season	Yellow cards	Red cards	Penalties
Premier League 2016/17	3.66 (± 0.24)	0.11 (± 0.05)	0.28 (± 0.14)
Championship 2016/17	3.55 (± 0.18)	0.17 (± 0.05)	0.28 (± 0.07)
Premier League 2017/18	3.06 (± 0.24)	0.1 (± 0.03)	0.21 (± 0.07)
Championship 2017/18	3.51 (± 0.24)	0.15 (± 0.04)	0.2 (± 0.04)
Premier League 2018/19	3.21 (± 0.28)	0.12 (± 0.04)	0.27 (± 0.08)
Championship 2018/19	3.44 (± 0.27)	0.14 (± 0.04)	0.24 (± 0.05)

The average number of yellow cards, red cards, and penalties per match in the Premier League and the Championship across the 2016/17, 2017/18, and 2018/19 seasons and will be used in the parallel trends assumption analysis.

For red cards, pre-intervention trends were broadly comparable. While the Championship showed a small but steady decline and the EPL remained stable before a modest rise in 2018/19, the overall patterns suggest moderate support for the identifying assumption.

For penalties, trends were highly consistent across leagues. Both the EPL and the Championship exhibited a decline from 2016/17 to 2017/18 followed by an increase in 2018/19, indicating strong support for the parallel trends assumption for this outcome.

For yellow cards, the two leagues displayed non-parallel trajectories. The Championship showed a gradual decline across the three seasons, whereas the EPL experienced a sharper decrease between 2016/17 and 2017/18 before rising slightly in 2018/19. This pattern suggests limited support for the parallel trends assumption for yellow cards, and results for this outcome are therefore interpreted more cautiously.

Descriptive statistics for the 2018/19 (without VAR) and 2023/24 (with VAR) seasons are summarized in Table 3 (see Appendix, Tables A1, B1, A2, and B2 for a complete overview of incidents by season/team). In the EPL, the mean number of yellow cards, red cards and penalties increased by 0.99, 0.03 and 0.01 per match, respectively. Over the same period, the Championship recorded an increase in yellow cards by 0.62 per match. However, contrary to the EPL, a decline in number of red cards (−0.04) and penalties (−0.06) per match were observed. These descriptive patterns suggest diverging developments between the two leagues following the introduction of VAR.

**Table 3:** Average variables per match.

League and season	Yellow cards	Red cards	Penalties
Premier League 2018/19	3.21 (± 0.28)	0.12 (± 0.04)	0.27 (±0.08)
Championship 2018/19	3.44 (± 0.27)	0.14 (± 0.04)	0.24 (± 0.05)
Premier League 2023/24	4.2 (± 0.35)	0.15 (± 0.04)	0.28 (± 0.08)
Championship 2023/24	4.06 (± 0.24)	0.1 (± 0.03)	0.18 (± 0.07)

This table presents the numbers of yellow cards, red cards, and penalties awarded per match in the Premier League and the Championship during the 2018/2019 and 2023/2024 seasons. These values serve as the basis for the Difference-in-Difference analysis.

### Difference-in-differences analysis

The results of the DiD analysis are summarized in Table 4. The estimated DiD effect for yellow cards is 0.37 per match (SE = 0.0277; 95% CI [0.3157, 0.4243]; *p* < 0.001). For red cards, the estimated effect is 0.07 per match (SE = 0.0036; 95% CI [0.0629, 0.0771]; *p* < 0.001). For penalties, the estimated DiD effect is 0.07 per match (SE = 0.0068; 95% CI [0.0566, 0.0834]; *p* < 0.001). Together, these estimates indicate that changes in disciplinary outcomes in the EPL differed from contemporaneous developments in the Championship over the study period. In line with the pre-intervention trend assessment, interpretation is strongest for penalties, more tentative for red cards, and most cautious for yellow cards.

**Table 4:** Summary of difference-in-difference estimates for yellow cards, red cards, and penalties.

Estimation results	Yellow cards (*n* = 1,864)	Red cards (*n* = 1,864)	Penalties (*n* = 1,864)
Estimated $\delta_{\mathrm{DD}}$	0.37	0.07	0.07
Standard error	0.0277	0.0036	0.0068
Confidence interval	0.4243: 0.3157	0.0771: 0.0629	0.0834: 0.0566
*t*-value	13.3580	19.2269	10.2497
*p*-value	*p* < 0.001	*p* < 0.001	*p* < 0.001

Estimated difference-in-difference effects for each outcome variable (yellow cards, red cards, and penalties), including standard errors, 95% confidence intervals, *t*-values, and *p*-values. All estimates are based on 1864 match-level observations from the Premier League and the Championship, comparing the 2018/19 (pre-VAR) and 2023/24 (post-VAR) seasons.

## Discussion

This study examined whether the implementation of the VAR in the English Premier League (EPL) was associated with changes in key disciplinary outcomes relative to contemporaneous developments in the English Championship. Conceptually, VAR can be understood as a rule-enforcement and governance innovation that reshapes decision-making conditions for officials through technological oversight and formalized review procedures (Nagle et al., 2024; Zglinski, 2020). Within this framing, the DiD estimates indicate relative increases in penalties and red cards, outcomes directly within VAR’s review scope (FIFA, 2018; Spitz et al., 2021). For yellow cards (not directly reviewable by VAR), a relative increase in yellow cards was observed. However, there were limitations for this outcome in the pre-trend assumption analysis. Therefore, findings related to yellow cards must be considered as exploratory. Overall, interpretation is therefore outcome-specific and should be calibrated to the plausibility of the identifying assumption, supported most strongly for penalties, moderately for red cards, and weakly for yellow cards.

Across outcomes, the DiD estimates represent differences in changes between leagues rather than simple within-league before–after differences. In football-specific terms, the estimated effects correspond to approximately 7 additional red cards and 7 additional penalties per 100 matches (0.07 per match), and approximately 37 additional yellow cards per 100 matches (0.37 per match), in the EPL relative to the contemporaneous change observed in the Championship. Scaled to a full EPL season (380 matches), this equates to approximately 27 additional red cards and penalties, and approximately 141 additional yellow cards, respectively. As descriptive benchmarks anchored to the EPL pre-VAR means (2018/19), these effects correspond to approximately 11.5% of the baseline yellow-card rate, 58.3% of the baseline red-card rate, and 25.9% of the baseline penalty rate. Notably, these proportions should be interpreted as scaling of the DiD estimates (differences in changes), not as simple within-league percentage increases.

Penalties provide the strongest support for interpretation within the DiD framework. First, pre-intervention penalty trajectories were highly consistent across leagues, strengthening the plausibility of the parallel trends assumption for this outcome. Second, penalty decisions fall squarely within VAR’s formal remit, and existing evidence indicates that VAR can improve accuracy in “game-changing” incidents (Spitz et al., 2021; FIFA, 2018). The present estimate, a relative increase of 0.07 penalties per match, aligns with research showing that VAR can alter the frequency of sanctioning in elite football and can mitigate certain forms of referee bias (Gasparetto and Loktionov, 2023). In the EPL context, where penalty decisions are both high-impact and publicly scrutinized (Scanlon et al., 2022), VAR may reduce the probability that a penalty-area infringement goes unpunished or that a penalty is incorrectly awarded, thereby shifting the net rate of penalties awarded.

For red cards, the DiD estimate suggests a relative increase of 0.07 red cards per match in the EPL compared with the Championship. Direct red cards are reviewable by VAR, and the estimate is therefore consistent with VAR facilitating the identification and correction of serious foul play or violent conduct that can be difficult to adjudicate in real time (FIFA, 2018; Spitz et al., 2021). At the same time, red cards are rare events, and pre-intervention trends were less tightly aligned than for penalties. Accordingly, interpretation should be treated with moderate caution, and the result is best understood as consistent with, rather than definitive evidence of, a VAR-related shift in sanctioning for the most severe incidents.

The relative increase in yellow cards should be interpreted most cautiously. Yellow cards are outside VAR’s direct review scope, and the pre-intervention trajectories were not parallel between the EPL and Championship, weakening the identifying assumption for this outcome. For that reason, the yellow card finding is best treated as exploratory and as a potential indicator of indirect behavioural and institutional effects rather than a direct technological effect. One plausible interpretation is that VAR changes the broader informational environment of officiating. Even when not directly reviewable, cautioning decisions may be shaped by perceived oversight, heightened accountability, and attempts to maintain game control under increased scrutiny (Nagle et al., 2024; Unkelbach and Memmert, 2008). From the players’ perspective, VAR can also catalyze behavioural adaptation and changes in how incidents are experienced and managed on the pitch (Archibald et al., 2025). However, alternative explanations remain credible, including league-specific changes in playing style, refereeing guidance, or threshold calibration over time. This uncertainty reinforces the need to treat yellow-card changes as hypothesis-generating rather than as a well-identified VAR effect.

### Positioning relative to prior VAR research

The present findings are broadly consistent with the view that VAR most clearly affects outcomes that fall within its formal protocol (FIFA, 2018; Spitz et al., 2021). At the same time, prior empirical studies have reported heterogeneous effects across leagues, World Cup and outcome measures (e.g., Han et al., 2020; Bao and Han, 2024; Kubayi et al., 2022; Lago-Peñas et al., 2019, 2021; Spitz et al., 2021). For example, Lago-Peñas et al. (2019) reported reductions in several match events (including yellow cards) in Serie A and the Bundesliga following VAR, whereas Errekagorri et al. (2020) documented changes in performance indicators and playing time in LaLiga. Such cross-league differences may reflect variation in implementation, competition culture, and governance (Han et al., 2020; Sarmento et al., 2013) supporting the argument that VAR functions as a socio-technical system whose effects emerge through interaction between technology, institutional rules, and human decision-makers (Archibald et al., 2025; Nagle et al., 2024). In the EPL, the introduction of VAR has also been accompanied by strong public debate and contested legitimacy, which can influence the social context in which referees operate (Bertin et al., 2023; Hamsund and Scelles, 2021; Scanlon et al., 2022).

### Limitations and implications for interpretation

A key contribution of the DiD approach is that it controls common time trends by benchmarking changes in the EPL against a comparable domestic league without VAR. Nevertheless, identification depends on the plausibility of parallel trends, and the diagnostic evidence indicates outcome-specific differences in credibility. In addition, the analysis relies on season-level comparisons and aggregated match averages. While this supports transparent estimation, it does not adjust for match-specific context or potential compositional changes in teams and playing styles. These considerations do not invalidate the observed estimates, but they motivate calibrated inference, particularly for outcomes with weaker pre-trend support. One limitation set in using a single season on each side increases sensitivity to season-specific shake up such rule interpretations, referee directives, or unusual competitive dynamics. Subsequently, such as season-specific shocks or institutional learning effects cannot be fully disentangled within the design of current study, and it must be noted that the findings reflect the traditional VAR regime in EPL rather than the immediate implementation phase. To avoid the potential influence from the COVID 19 pandemic we chose the season before (2018/19) and after (2023/24). Nevertheless, one must take into consideration that both these seasons may have been subject to various influences such as the use of new technology and/or the after-effects of extensive restrictions in daily life.

### Practical implications and future research

From a practical perspective, the results suggest that the established VAR regime in the EPL is associated with meaningful changes in the frequency of penalties and red cards at competition scale, with potential implications for competitive balance, deterrence, and perceptions of fairness (Tamir and Bar-Eli, 2021). However, increased VAR interventions and -review can also impose costs through disruption of match flow and delays, which has been highlighted as a key concern in both empirical work and fan discourse (Errekagorri et al., 2020; Scanlon et al., 2022). Future research should therefore move beyond sanction counts to integrate officiating outcomes with measures of review frequency and duration and should evaluate how VAR shapes decision thresholds and referee behaviour over time. Designs incorporating multiple post-VAR seasons and richer match-level covariates would strengthen inference and help differentiate improved accuracy from shifting enforcement norms.

## Conclusion

This study investigated the impact of VAR implementation on officiating within the EPL. Utilizing a DiD framework with the Championship as a control group, the analysis reveals that the established VAR regime is associated with a statistically significant increase in red cards and penalties. As both metrics involve direct VAR intervention, these findings suggest that the technology facilitates more rigorous rule enforcement and potentially enhances the correction of clear and obvious errors. The observed rise in yellow cards may indicate indirect behavioural shifts but need to be cautiously interpreted. Future research should examine the long-term psychological effects of VAR on referees’ subsequent decision-making and provide comparative analyses across international domestic leagues.

## Data Availability

The original contributions presented in the study are included in the article/Supplementary material, further inquiries can be directed to the corresponding author.
