# Supplementary material for: When technology meets judgment: outcome of football referees’ disciplinary decision-making after the implementation of VAR in the English Premier League
Source: Front Psychol. 2026 Mar 19;17:1769008. doi: 10.3389/fpsyg.2026.1769008 (PMC13044101; doi:10.3389/fpsyg.2026.1769008)
Supplement: Supplementary file 1 [file Data_Sheet_1.docx]

Appendix

This appendix includes tables used in the study. Tables A1 and B1 present team-level data from the Premier League (2018/19 and 2023/24), while Tables A2 and B2 show corresponding data from the Championship. Tables A3 and B3 provide aggregated values used to calculate the statistical estimates in the Difference-in-Differences analysis. Tables A4, B4, and C4 illustrate graphically assessment of the parallel trends assumption used for yellow cards, red cards, and penalties respectively.

**Table A1.** Premier League team statistics (2018/19 Season)

| **Teams** | **Yellow cards** | **Red Cards** | **Penalties awarded** | **Matches Played** |
| --- | --- | --- | --- | --- |
|  |  |  |  |  |
| Manchester City | 44 | 1 | 4 | 38 |
| Liverpool | 37 | 2 | 7 | 38 |
| Chelsea | 49 | 0 | 5 | 38 |
| Tottenham | 56 | 3 | 4 | 38 |
| Arsenal | 72 | 2 | 5 | 38 |
| Manchester United | 73 | 4 | 12 | 38 |
| Wolverhampton | 72 | 1 | 4 | 38 |
| Everton | 55 | 4 | 5 | 38 |
| Leicester | 57 | 5 | 7 | 38 |
| West Ham United | 59 | 1 | 5 | 38 |
| Watford | 77 | 4 | 1 | 38 |
| Crystal Palace | 58 | 2 | 11 | 38 |
| Newcastle | 57 | 2 | 3 | 38 |
| Bournemouth | 60 | 1 | 9 | 38 |
| Burnley | 75 | 1 | 2 | 38 |
| Southampton | 71 | 3 | 5 | 38 |
| Brighton | 60 | 4 | 6 | 38 |
| Cardiff | 66 | 1 | 4 | 38 |
| Fulham | 68 | 2 | 3 | 38 |
| Huddersfield | 55 | 4 | 1 | 38 |

***Table A1:*** Yellow cards, red cards, penalties awarded, and matches played by Premier League teams in the 2018/19 season

**Table B1.** Premier League team statistics (2023/24 Season)

***Table B1:*** Yellow cards, red cards, penalties awarded, and matches played by Premier League teams in the 2023/24 season.

| **Teams** | **Yellow cards** | **Red Cards** | **Penalties awarded** | **Matches Played** |
| --- | --- | --- | --- | --- |
|  |  |  |  |  |
| Manchester City | 52 | 2 | 10 | 38 |
| Arsenal | 62 | 2 | 10 | 38 |
| Liverpool | 65 | 5 | 9 | 38 |
| Aston Villa | 94 | 2 | 4 | 38 |
| Tottenham | 89 | 4 | 2 | 38 |
| Chelsea | 105 | 4 | 12 | 38 |
| Newcastle | 72 | 1 | 9 | 38 |
| Manchester United | 81 | 1 | 7 | 38 |
| West Ham United | 76 | 3 | 5 | 38 |
| Crystal Palace | 69 | 2 | 4 | 38 |
| Brighton | 91 | 3 | 6 | 38 |
| Bournemouth | 79 | 3 | 3 | 38 |
| Fullham | 78 | 4 | 2 | 38 |
| Wolverhampton | 100 | 4 | 4 | 38 |
| Everton | 80 | 1 | 3 | 38 |
| Brentford | 89 | 2 | 3 | 38 |
| Nottingham Forrest | 78 | 3 | 1 | 38 |
| Luton | 71 | 0 | 5 | 38 |
| Burnley | 71 | 7 | 3 | 38 |
| Sheffield United | 95 | 5 | 5 | 38 |

**Table A2.** Championship team statistics (2018/19 season)

| **Teams** | **Yellow cards** | **Red Cards** | **Penalties awarded** | **Matches Played** |
| --- | --- | --- | --- | --- |
|  |  |  |  |  |
| Norwich | 79 | 1 | 7 | 46 |
| Sheffield United | 75 | 3 | 7 | 46 |
| Leeds | 91 | 5 | 5 | 46 |
| West Bromwich Albion | 82 | 7 | 9 | 46 |
| Aton Villa | 83 | 3 | 8 | 46 |
| Derby County | 106 | 2 | 5 | 46 |
| Middlesbrough | 79 | 3 | 5 | 46 |
| Bristol City | 80 | 2 | 2 | 46 |
| Nottingham Forrest | 104 | 6 | 9 | 46 |
| Swansea | 45 | 1 | 8 | 46 |
| Brentford | 74 | 3 | 3 | 46 |
| Sheffield Wednesday | 76 | 3 | 4 | 46 |
| Hull City | 69 | 1 | 5 | 46 |
| Preston North End | 69 | 7 | 5 | 46 |
| Blackburn Rovers | 73 | 2 | 9 | 46 |
| Stoke City | 71 | 5 | 8 | 46 |
| Birmingham | 81 | 3 | 3 | 46 |
| Wigan Athletic | 91 | 4 | 9 | 46 |
| Queens Park Rangers | 92 | 1 | 5 | 46 |
| Reading | 81 | 3 | 4 | 46 |
| Millwall | 78 | 1 | 5 | 46 |
| Rotherham United | 71 | 3 | 7 | 46 |
| Bolton Wanders | 75 | 4 | 1 | 46 |
| Ipswich | 74 | 5 | 2 | 46 |

***Table A2:*** Yellow cards, red cards, penalties awarded, and matches played by Championship teams in the 2018/19 season.

**Table B2.** Championship team statistics (2023/24 season)

| **Teams** | **Yellow cards** | **Red Cards** | **Penalties awarded** | **Matches Played** |
| --- | --- | --- | --- | --- |
|  |  |  |  |  |
| Leicester | 86 | 1 | 13 | 46 |
| Ipswich | 101 | 0 | 3 | 46 |
| Leeds | 77 | 2 | 9 | 46 |
| Southampton | 111 | 3 | 5 | 46 |
| West Bromwich Albion | 84 | 1 | 5 | 46 |
| Norwich | 90 | 2 | 3 | 46 |
| Hull City | 101 | 1 | 7 | 46 |
| Middlesborough | 83 | 2 | 5 | 46 |
| Coventry City | 90 | 3 | 6 | 46 |
| Preston North End | 110 | 1 | 3 | 46 |
| Bristol City | 71 | 1 | 7 | 46 |
| Cardiff | 80 | 1 | 2 | 46 |
| Millwall | 105 | 2 | 2 | 46 |
| Swansea | 90 | 4 | 7 | 46 |
| Watford | 100 | 3 | 1 | 46 |
| Sunderland | 91 | 2 | 6 | 46 |
| Stoke City | 108 | 3 | 4 | 46 |
| Queens Park Rangers | 98 | 4 | 2 | 46 |
| Black Burn Rovers | 93 | 5 | 1 | 46 |
| Sheffield Wednesday | 104 | 5 | 0 | 46 |
| Plymouth | 100 | 2 | 1 | 46 |
| Birmingham | 100 | 3 | 3 | 46 |
| Huddersfield Town | 87 | 2 | 1 | 46 |
| Rotherham | 82 | 4 | 2 | 46 |

***Table B2:*** Yellow cards, red cards, penalties awarded, and matches played by Championship teams in the 2023/24 season.

**Table A3**. Match Statistics per team in the Premier League and Championship (2018/19)

| **League** | **Season** | **Team** | **Yellow Cards per match** | **Red cards per match** | **Penalties per match** |
| --- | --- | --- | --- | --- | --- |
| Premier League | 2018/19 | Manchester City | 1.1579 | 0.0263 | 0.1053 |
| Premier League | 2018/19 | Liverpool | 0.9737 | 0.0526 | 0.1842 |
| Premier League | 2018/19 | Chelsea | 1.2895 | 0.0000 | 0.1316 |
| Premier League | 2018/19 | Tottenham | 1.4737 | 0.0789 | 0.1053 |
| Premier League | 2018/19 | Arsenal | 1.8947 | 0.0526 | 0.1316 |
| Premier League | 2018/19 | Manchester United | 1.9211 | 0.1053 | 0.3158 |
| Premier League | 2018/19 | Wolverhampton | 1.8947 | 0.0263 | 0.1053 |
| Premier League | 2018/19 | Everton | 1.4474 | 0.1053 | 0.1316 |
| Premier League | 2018/19 | Leicester | 1.5000 | 0.1316 | 0.1842 |
| Premier League | 2018/19 | West Ham United | 1.5526 | 0.0263 | 0.1316 |
| Premier League | 2018/19 | Watford | 2.0263 | 0.1053 | 0.0263 |
| Premier League | 2018/19 | Crystal Palace | 1.5263 | 0.0526 | 0.2895 |
| Premier League | 2018/19 | Newcastle | 1.5000 | 0.0526 | 0.0789 |
| Premier League | 2018/19 | Bournemouth | 1.5789 | 0.0263 | 0.2368 |
| Premier League | 2018/19 | Burnley | 1.9737 | 0.0263 | 0.0526 |
| Premier League | 2018/19 | Southampton | 1.8684 | 0.0789 | 0.1316 |
| Premier League | 2018/19 | Brighton | 1.5789 | 0.1053 | 0.1579 |
| Premier League | 2018/19 | Cardiff | 1.7368 | 0.0263 | 0.1053 |
| Premier League | 2018/19 | Fulham | 1.7895 | 0.0526 | 0.0789 |
| Premier League | 2018/19 | Huddersfield | 1.4474 | 0.1053 | 0.0263 |
| Championship | 2018/19 | Norwich | 1.7174 | 0.0217 | 0.1522 |
| Championship | 2018/19 | Sheffield United | 1.6304 | 0.0652 | 0.1522 |
| Championship | 2018/19 | Leeds | 1.9783 | 0.1087 | 0.1087 |
| Championship | 2018/19 | West Bromwich Albion | 1.7826 | 0.1522 | 0.1957 |
| Championship | 2018/19 | Aton Villa | 1.8043 | 0.0652 | 0.1739 |
| Championship | 2018/19 | Derby County | 2.3043 | 0.0435 | 0.1087 |
| Championship | 2018/19 | Middlesbrough | 1.7174 | 0.0652 | 0.1087 |
| Championship | 2018/19 | Bristol City | 1.7391 | 0.0435 | 0.0435 |
| Championship | 2018/19 | Nottingham Forrest | 2.2609 | 0.1304 | 0.1957 |
| Championship | 2018/19 | Swansea | 0.9783 | 0.0217 | 0.1739 |
| Championship | 2018/19 | Brentford | 1.6087 | 0.0652 | 0.0652 |
| Championship | 2018/19 | Sheffield Wednesday | 1.6522 | 0.0652 | 0.0870 |
| Championship | 2018/19 | Hull City | 1.5000 | 0.0217 | 0.1087 |
| Championship | 2018/19 | Preston North End | 1.5000 | 0.1522 | 0.1087 |
| Championship | 2018/19 | Blackburn Rovers | 1.5870 | 0.0435 | 0.1957 |
| Championship | 2018/19 | Stoke City | 1.5435 | 0.1087 | 0.1739 |
| Championship | 2018/19 | Birmingham | 1.7609 | 0.0652 | 0.0652 |
| Championship | 2018/19 | Wigan Athletic | 1.9783 | 0.0870 | 0.1957 |
| Championship | 2018/19 | Queens Park Rangers | 2.0000 | 0.0217 | 0.1087 |
| Championship | 2018/19 | Reading | 1.7609 | 0.0652 | 0.0870 |
| Championship | 2018/19 | Millwall | 1.6957 | 0.0217 | 0.1087 |
| Championship | 2018/19 | Rotherham United | 1.5435 | 0.0652 | 0.1522 |
| Championship | 2018/19 | Bolton Wanders | 1.6304 | 0.0870 | 0.0217 |
| Championship | 2018/19 | Ipswich | 1.6087 | 0.1087 | 0.0435 |

***Table A3:*** Average number of yellow cards, red cards, and penalties awarded per match for each team in the Premier League and the Championship during the 2018/19 season. These values were used in the calculation of Difference-in-Difference estimates.

**Table B3**. Match Statistics per team in the Premier League and Championship (2023/24)

| **League** | **Season** | **Team** | **Yellow Cards per match** | **Red cards per match** | **Penalties per match** |
| --- | --- | --- | --- | --- | --- |
| Premier League | 2023/24 | Manchester City | 1.3684 | 0.0526 | 0.2632 |
| Premier League | 2023/24 | Arsenal | 1.6316 | 1.6316 | 0.2632 |
| Premier League | 2023/24 | Liverpool | 1.7105 | 1.7105 | 0.2368 |
| Premier League | 2023/24 | Aston Villa | 2.4737 | 0.0526 | 0.1053 |
| Premier League | 2023/24 | Tottenham | 2.3421 | 2.3421 | 0.0526 |
| Premier League | 2023/24 | Chelsea | 2.7632 | 2.7632 | 0.3158 |
| Premier League | 2023/24 | Newcastle | 1.8947 | 0.0263 | 0.2368 |
| Premier League | 2023/24 | Manchester United | 2.1316 | 2.1316 | 0.1842 |
| Premier League | 2023/24 | West Ham United | 2.0000 | 2.0000 | 0.1316 |
| Premier League | 2023/24 | Crystal Palace | 1.8158 | 0.0526 | 0.1053 |
| Premier League | 2023/24 | Brighton | 2.3947 | 2.3947 | 0.1579 |
| Premier League | 2023/24 | Bournemouth | 2.0789 | 2.0789 | 0.0789 |
| Premier League | 2023/24 | Fullham | 2.0526 | 0.1053 | 0.0526 |
| Premier League | 2023/24 | Wolverhampton | 2.6316 | 2.6316 | 0.1053 |
| Premier League | 2023/24 | Everton | 2.1053 | 2.1053 | 0.0789 |
| Premier League | 2023/24 | Brentford | 2.3421 | 0.0526 | 0.0789 |
| Premier League | 2023/24 | Nottingham Forrest | 2.0526 | 2.0526 | 0.0263 |
| Premier League | 2023/24 | Luton | 1.8684 | 1.8684 | 0.1316 |
| Premier League | 2023/24 | Burnley | 1.8684 | 0.1842 | 0.0789 |
| Premier League | 2023/24 | Sheffield United | 2.5000 | 2.5000 | 0.1316 |
| Championship | 2023/24 | Leicester | 1.8696 | 0.0217 | 0.2826 |
| Championship | 2023/24 | Ipswich | 2.1957 | 0.0000 | 0.0652 |
| Championship | 2023/24 | Leeds | 1.6739 | 0.0435 | 0.1957 |
| Championship | 2023/24 | Southampton | 2.4130 | 0.0652 | 0.1087 |
| Championship | 2023/24 | West Bromwich Albion | 1.8261 | 0.0217 | 0.1087 |
| Championship | 2023/24 | Norwich | 1.9565 | 0.0435 | 0.0652 |
| Championship | 2023/24 | Hull City | 2.1957 | 0.0217 | 0.1522 |
| Championship | 2023/24 | Middlesborough | 1.8043 | 0.0435 | 0.1087 |
| Championship | 2023/24 | Coventry City | 1.9565 | 0.0652 | 0.1304 |
| Championship | 2023/24 | Preston North End | 2.3913 | 0.0217 | 0.0652 |
| Championship | 2023/24 | Bristol City | 1.5435 | 0.0217 | 0.1522 |
| Championship | 2023/24 | Cardiff | 1.7391 | 0.0217 | 0.0435 |
| Championship | 2023/24 | Millwall | 2.2826 | 0.0435 | 0.0435 |
| Championship | 2023/24 | Swansea | 1.9565 | 0.0870 | 0.1522 |
| Championship | 2023/24 | Watford | 2.1739 | 0.0652 | 0.0217 |
| Championship | 2023/24 | Sunderland | 1.9783 | 0.0435 | 0.1304 |
| Championship | 2023/24 | Stoke City | 2.3478 | 0.0652 | 0.0870 |
| Championship | 2023/24 | Queens Park Rangers | 2.1304 | 0.0870 | 0.0435 |
| Championship | 2023/24 | Black Burn Rovers | 2.0217 | 0.1087 | 0.0217 |
| Championship | 2023/24 | Sheffield Wednesday | 2.2609 | 0.1087 | 0.0000 |
| Championship | 2023/24 | Plymouth | 2.1739 | 0.0435 | 0.0217 |
| Championship | 2023/24 | Birmingham | 2.1739 | 0.0652 | 0.0652 |
| Championship | 2023/24 | Huddersfield Town | 1.8913 | 0.0435 | 0.0217 |
| Championship | 2023/24 | Rotherham | 1.7826 | 0.0870 | 0.0435 |

***Table B3:*** Average number of yellow cards, red cards, and penalties awarded per match for each team in the Premier League and the Championship during the 2023/24 season. These values were used in the calculation of Difference-in-Difference estimates.

**Graphical Assessment of the Parallel Trends Assumption**

**Figure A4.** Parallel trends assumption for yellow cards

***Figure A4:*** Illustrates the average number of yellow cards per match in the Premier League and the Championship over the 2016/17, 2017/18 and 2018/19 seasons.

**Figure B4.** Parallel trend assumption for red cards

***Figure B4:*** illustrates the average number of red cards per match in the Premier League and the Championship from the 2016/17, 2017/18 and 2018/19 seasons.

**Figure C4.** Parallel trend assumption for penalties

***Figure C4:*** illustrates the average number of penalties awarded per match in the Premier League and the Championship from the 2016/17, 2017/18 and 2018/19 seasons.
